# CTCF Mediates Replicative Senescence Through POLD1

**DOI:** 10.3389/fcell.2021.618586

**Published:** 2021-02-22

**Authors:** Yuli Hou, Qiao Song, Shichao Gao, Xiaomin Zhang, Yaqi Wang, Jing Liu, Jingxuan Fu, Min Cao, Peichang Wang

**Affiliations:** Clinical Laboratory of Xuanwu Hospital, Capital Medical University, Beijing, China

**Keywords:** POLD1, transcription factor, CTCF, aging, transcriptional regulation

## Abstract

POLD1, the catalytic subunit of DNA polymerase δ, plays a critical role in DNA synthesis and DNA repair processes. Moreover, POLD1 is downregulated in replicative senescence to mediate aging. In any case, the components of age-related downregulation of POLD1 expression have not been fully explained. In this article, we elucidate the mechanism of the regulation of POLD1 at the transcription level and found that the transcription factor CCCTC-binding factor (CTCF) was bound to the POLD1 promoter area in two sites. The binding level of CTCF for the POLD1 promoter appeared to be related to aging and was confirmed to be positively controlled by the CTCF level. Additionally, cell senescence characteristics were detected within the cells transfected with short hairpin RNA (shRNA)-CTCF, pLenti-CMV-CTCF, shRNA-POLD1, and pLenti-CMV-POLD1, and the results showed that the CTCF may contribute to the altered expression of POLD1 in aging. In conclusion, the binding level of CTCF for the POLD1 promoter intervened by an age-related decrease in CTCF and downregulated the POLD1 expression in aging. Moreover, the decrease in CTCF-mediated POLD1 transcription accelerates the progression of cell aging.

## Introduction

Aging is the major risk factor for most chronic diseases that account for the bulk of morbidity, mortality, and health costs in developed and developing countries (Harper, 2014; Childs et al., 2015; Kirkland and Tchkonia, 2017). There are various theories on the occurrence of aging (Da et al., 2016), and one of the theories is genomic instability and decreased proliferation ability, which is considered a major driving force of aging (Sun et al., 2018; Hou et al., 2019). POLD1, as the catalytic subunit of DNA polymerase delta and endowed with polymerase and exonuclease activities, plays a critical role in DNA synthesis and DNA repair processes (Cui et al., 2019). Our previous study found that POLD1 expression was downregulated as cell aging in human lymphocytes and preliminarily confirmed that the expression of POLD1 was essential in cell cycle regulation and DNA damage repair processes (Wang et al., 2012; Song et al., 2015; Maierhofer et al., 2019). However, the mechanism of age-related downregulation of POLD1 expression has not been fully explained.

Clearly, transcriptional regulation is the main regulatory mechanism of gene expression (Tepper et al., 2013), and several defined transcription factors (TFs) can bind to the POLD1 promoter to regulate cell cycle and aging (Li and Lee, 2001; Song et al., 2009). Identifying the POLD1 associated with other TFs is important to understand the mechanism of the regulation of POLD1 in aging. The CCCTC-binding factor (CTCF) TF, which can bind to DNA sequences or proteins through its special 11 zinc finger structure that forms CTCF–DNA and CTCF–protein complexes to regulate gene expression, plays an essential role in cell growth and differentiation (Chang et al., 2010; Song and Kim, 2017; Peng et al., 2019). Besides, many studies have shown that CTCF can bind to other key aging genes to regulate cell aging progress (Wendt et al., 2008). Therefore, the correlation between CTCF and POLD1 is worth exploring.

Previous studies have indicated that POLD1 and CTCF are closely related to cell growth and differentiation. In this study, CTCF was found to be one of the key TFs of POLD1, and it was hypothesized that the attenuation of the binding level of CTCF to the POLD1 promoter, mediated by an age-related decline in CTCF, downregulated POLD1 expression and accelerated replicative senescence.

## Results

### CTCF Mainly Binds to the POLD1 Promoter in Two Sites

**FIGURE 1:**
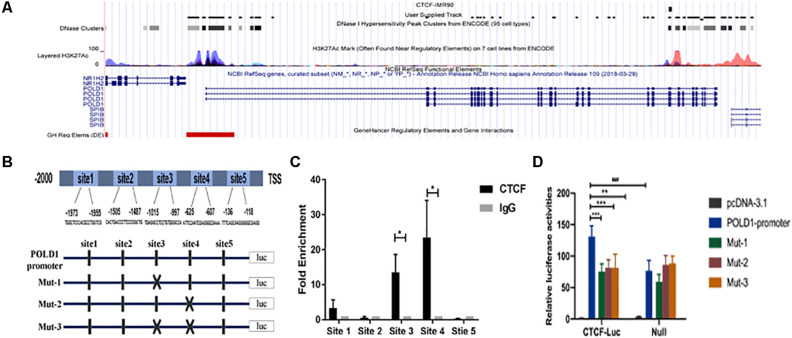
CCCTC-binding factor (CTCF) mainly binds to the DNA polymerase delta 1, catalytic subunit (POLD1) promoter in two sites. **(A)** ENCODE database analysis showing potential CTCF-binding sites in the POLD1 promoter. **(B)** The JASPAR database predicted five higher score binding sites of CTCF to the POLD1 promoter, and the mutations of the two CTCF-binding sites were designed. **(C)** The binding sites of CTCF to the POLD1 promoter were analyzed by chromatin immunoprecipitation (ChIP) assay in lymphocyte cells (*n* = 40). *P < 0.05 vs. IgG-negative control group. **(D)** Luciferase activity assay demonstrated that the CTCF led to an increase in POLD1 promoter activity and that mutation of site 3 or 4 greatly reduced promoter activity in HEK293T cells (*n* = 3). **P < 0.01, ***P < 0.005 vs. POLD1 promoter wild type (WT) + CTCF. ^###^*P* < 0.001 vs. POLD1 promoter WT. Data were analyzed using Student’s *t*-test, and data are shown as mean ± SEM, with three independent experiments in each group.

We analyzed the Encyclopedia of DNA Elements (ENCODE) database and found several transcription factor binding sites (TFBSs) of CTCF in the POLD1 promoter (Figure 1A). Potential specific CTCF-binding sites were analyzed with the assistance of the JASPAR database, and the results showed five potential CTCF-binding sites in the promoter of POLD1 with a higher score (Figure 1B). To directly determine which site CTCF binds to the POLD1 promoter, chromatin immunoprecipitation (ChIP)-qPCR assays were performed in lymphocyte cells. The ChIP results demonstrated that CTCF binds mainly to the POLD1 promoter in site 3 (−1015 to −997) and site 4 (−625 to −607) regions (Figure 1C). Evidence for transcriptional activation in a dual-luciferase reporter system showed that the region from the POLD1 promoter containing all sites displayed increased reporter activity relative to the control vector. Interestingly, mutation of site 3 or site 4 greatly reduced promoter activity, demonstrating that these are functional CTCF-binding sites in the human POLD1 promoter (Figure 1D).

### Both CTCF and POLD1 Expression Levels Were Reduced With Aging

**FIGURE 2:**
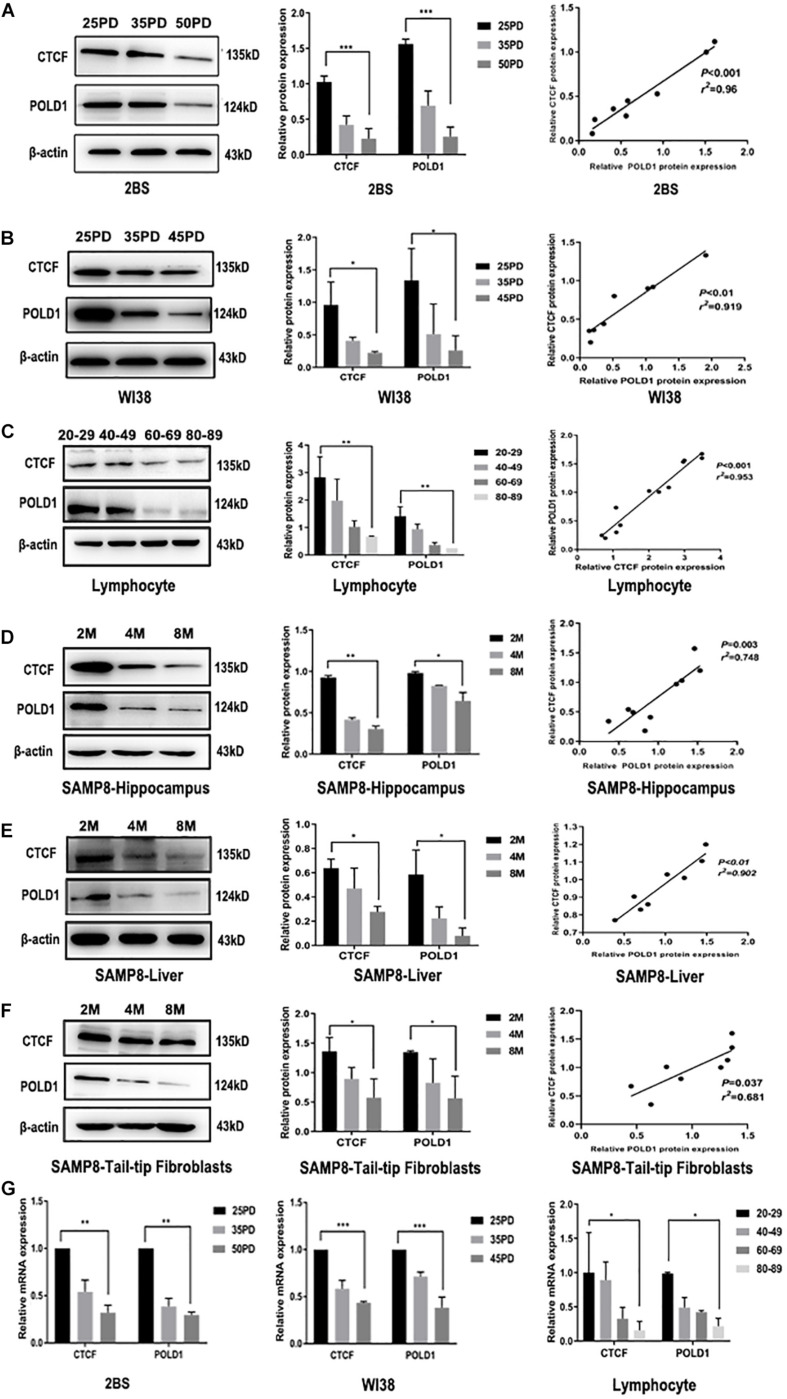
Both CCCTC-binding factor (CTCF) and DNA polymerase delta 1, catalytic subunit (POLD1) expression levels were reduced with aging. **(A)** Protein expression levels and relationship of CTCF and POLD1 in different population doublings (PDs) of 2BS cells (*n* = 3). **(B)** Protein expression levels and relationship of CTCF and POLD1 in different PDs of WI38 cells (*n* = 3). **(C)** Protein expression levels of CTCF and POLD1 in the lymphocytes of healthy people of different ages (*n* = 20 in every group). **(D)** Protein expression levels of CTCF and POLD1 in the hippocampus of SAMP8 mice at different ages in months (*n* = 18). **(E)** Protein expression levels of CTCF and POLD1 in livers of SAMP8 mice at different ages in months (*n* = 18). **(F)** Protein expression levels of CTCF and POLD1 in the 3PD mouse tail-tip fibroblasts of SAMP8 mice at different age in months (*n* = 18). **(G)** mRNA expression levels of CTCF and POLD1 in different PDs of 2BS, WI38 cells, and lymphocytes of healthy people of different ages. β-actin was used for the normalization of data. Data were analyzed using one-way ANOVA, and data are shown as mean ± SEM, with three independent experiments in each group. *P < 0.05, **P < 0.01, ***P < 0.005. The correlation was determined using Pearson’s correlation coefficient.

The expression levels of POLD1 and CTCF were detected in different population doublings (PDs) of human embryonic lung diploid fibroblasts (2BS) (25, 35, and 50 PDs) and human fetal lung fibroblast (WI38) (25, 35, and 45 PDs), lymphocytes of healthy people of different ages (20–29, 40–49, 60–69, and 80–89 years), and senescence-accelerated mouse prone (SAMP8) mice (Chen et al., 2019) at different ages in months (2, 4, and 8 months). The results showed that the protein expression levels of both CTCF and POLD1 were reduced consistently with aging, and there was a positive correlation between CTCF and POLD1 expression (Figures 2A–F). A similar result was found at the mRNA level (Figure 2G). These results indicate that reduced CTCF level could be responsible for the downregulation of POLD1.

### Binding Level of CTCF in the POLD1 Promoter Decreased With Aging

**FIGURE 3:**
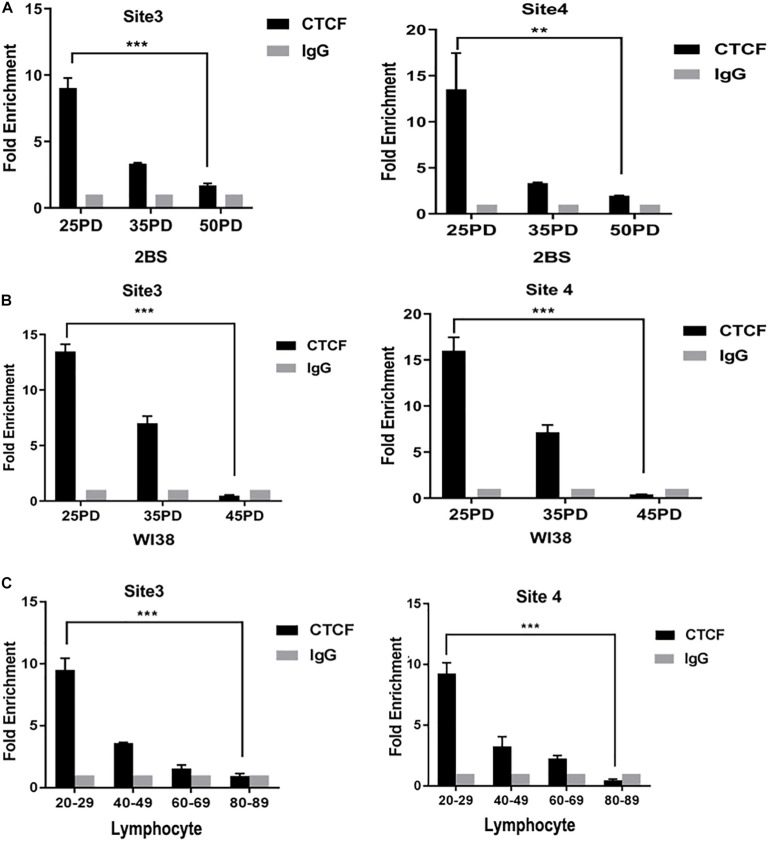
The binding level of CCCTC-binding factor (CTCF) in the DNA polymerase delta 1, catalytic subunit (POLD1) promoter decreased with aging. **(A)** Binding level of CTCF at the POLD1 promoter in 2BS cells from different population doublings (PDs). **(B)** Binding level of CTCF at the POLD1 promoter in WI38 cells from different PDs. **(C)** Binding level of CTCF at the POLD1 promoter in lymphocytes from healthy people of different ages. Densitometric analysis of the band intensity in different groups, normalized to the input. Data were compared using one-way ANOVA, and values represent the mean ± SEM with three independent experiments in each group. **P < 0.01, ***P < 0.005.

To further explore the change in the binding level of CTCF to POLD1 promoter, the binding level of CTCF in POLD1 promoter was detected in different PDs of 2BS and WI38 cells and in the lymphocytes of healthy people of different ages. The results showed that the binding levels of CTCF to the POLD1 promoter at sites 3 and 4 were both markedly decreased with aging in 2BS cell, WI38 cell, and lymphocytes (Figures 3A–C). These results suggested that the reduced level of CTCF could be related to the decreased binding level of CTCF to the POLD1 promoter to downregulate the expression of POLD1.

### CTCF Upregulated POLD1 Expression by Elevating the Binding Level of CTCF to the POLD1 Promoter

**FIGURE 4:**
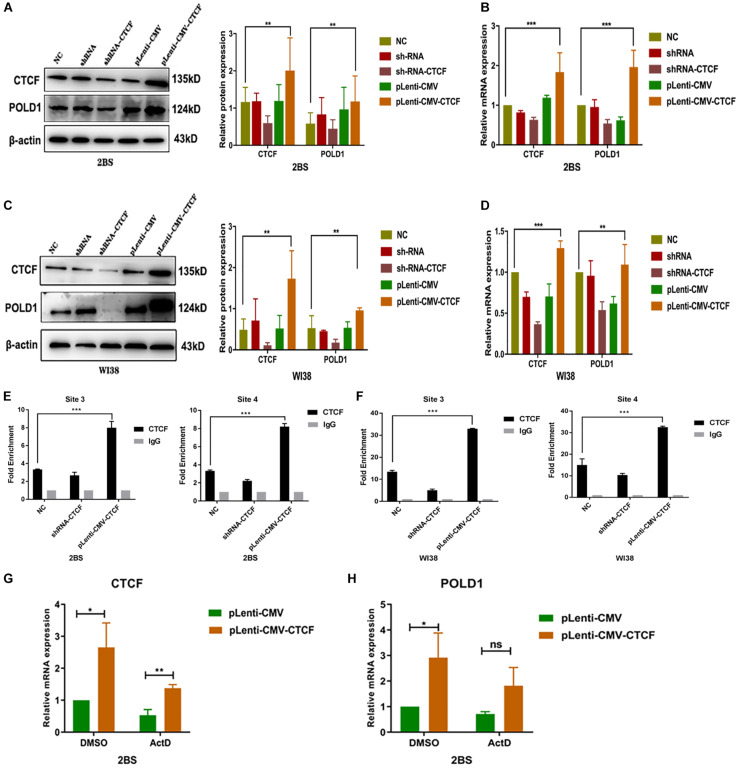
CCCTC-binding factor (CTCF) upregulated DNA polymerase delta 1, catalytic subunit (POLD1) expression by elevating the binding level of CTCF to the POLD1 promoter. **(A,C)** Protein expression levels of CTCF and POLD1 in 2BS **(A)** and WI38 cells **(C)**. **(B,D)** mRNA expression levels of CTCF and POLD1 in 2BS **(B)** and WI38 cells **(D)**. **(E,F)** Site 3 and 4 binding levels of CTCF at the POLD1 promoter in 2BS **(E)** and WI38 cells **(F)** transfected with short hairpin RNA (shRNA) and CTCF overexpression lentivirus. **(G,H)** CTCF **(G)** and POLD1 **(H)** mRNA levels in 2BS cells transfected with pLenti-CMV-CTCF lentivirus. Data were compared by one-way ANOVA and Student’s *t*-test, and data are shown as the mean ± SEM, with three independent experiments in each group. *P < 0.05, **P < 0.01, ***P < 0.005.

To illustrate the biological function of CTCF in POLD1 gene regulation, RNA interference (RNAi) and overexpression methods were used to regulate CTCF expression in 30 PD 2BS and 28 PD WI38 cells. The protein and mRNA expression levels of POLD1 and CTCF were determined by Western blot and quantitative reverse transcription-polymerase chain reaction (RT-qPCR), respectively. The results showed that the expression of CTCF and POLD1 was dramatically downregulated after transfection with short hairpin RNA (shRNA)-CTCF and upregulated after transfection with the CTCF eukaryotic expression lentivirus vector (Figures 4A–D). Furthermore, the binding level of CTCF to the POLD1 promoter at sites 3 and 4 in cells transfected with shRNA-CTCF was slightly lower than that of the control cells, and the binding level was higher in cells transfected with pLenti-CMV-CTCF than in the control cells (Figures 4E,F).

These results indicate that CTCF regulates POLD1 gene expression through transcription. To further verify this speculation, actinomycin D, a transcriptional inhibitor, was used. The results showed that the POLD1 upregulation induced by CTCF overexpression was blocked by actinomycin D (10 μg/ml) (Figures 4G,H).

### POLD1 Could Rescue the Effect of CTCF on POLD1 Expression

**FIGURE 5:**
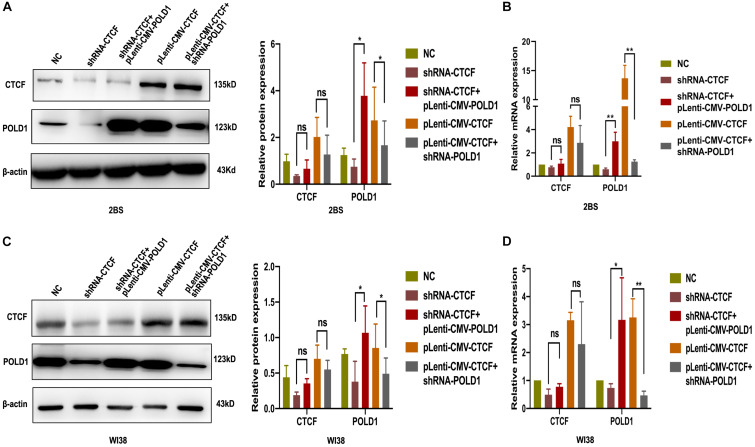
DNA polymerase delta 1, catalytic subunit (POLD1) rescued the effect of CCCTC-binding factor (CTCF) on POLD1 expression. **(A,C)** Protein expression of POLD1 and CTCF in 2BS **(A)** and WI38 cells **(C)**. **(B,D)** mRNA expression of POLD1 and CTCF in 2BS **(B)** and WI38 **(D)** cells. Data were compared by Student’s *t*-test, and data are shown as the mean ± SEM, with three independent experiments in each group. *P < 0.05, **P < 0.01.

Given that CTCF could affect the expression of POLD1, we tested whether overexpression or knockdown of POLD1 could interfere with the effect of CTCF on the POLD1 gene. shRNA-CTCF and pLenti-CMV-POLD1 were cotransfected into 2BS and WI38 cells, and pLenti-CMV-CTCF and shRNA-POLD1 were cotransfected into 2BS and WI38 cells. The protein expression and mRNA levels of CTCF and POLD1 were confirmed by Western blot and RT-qPCR, respectively. Upregulated expression of POLD1 was found in cells with shRNA-CTCF + pLenti-CMV-POLD1 than with shRNA-CTCF, and decreased POLD1 expression was observed in cells transfected with pLenti-CMV-CTCF + shRNA-POLD1 than with pLenti-CMV-CTCF (Figures 5A–D). These results showed that the POLD1 gene could rescue the effect of CTCF on the expression of POLD1.

### CTCF Was Involved in the Progression of Cellular Senescence, and POLD1 Could Rescue the Effect of Downregulated CTCF on Aging

**FIGURE 6:**
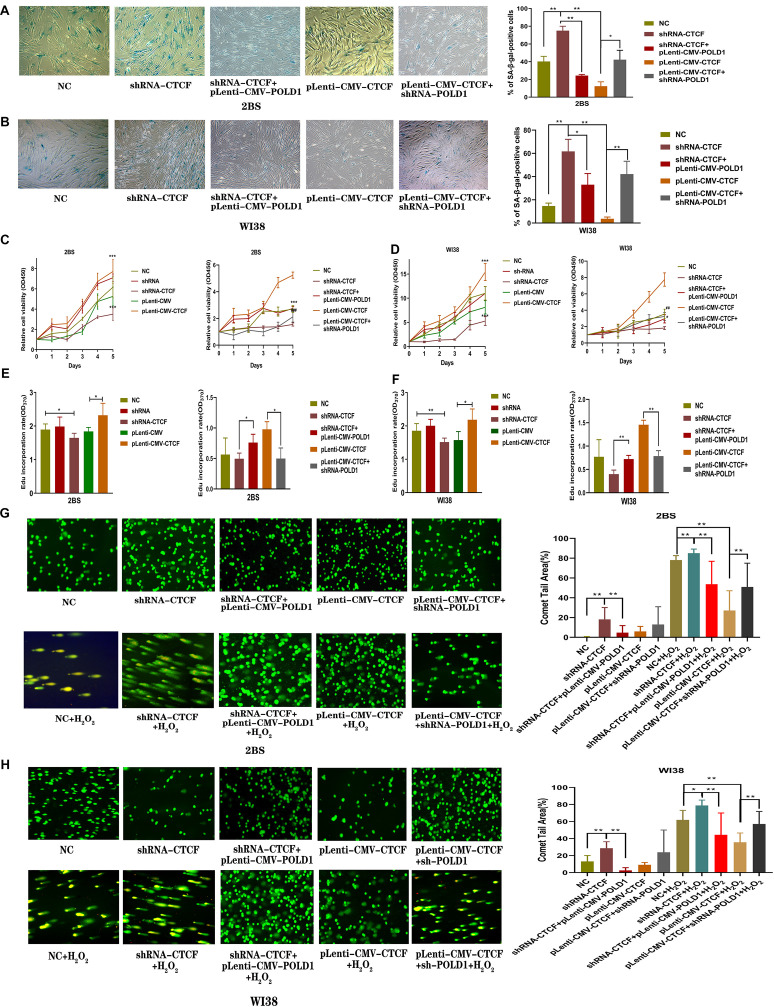
CCCTC-binding factor (CTCF) was involved in the progression of cellular senescence, and DNA polymerase delta 1, catalytic subunit (POLD1) rescued the effect of the downregulated CTCF on aging. **(A,B)** Senescence-associated β-galactosidase (SA-β-gal) staining of 2BS **(A)** and WI38 cells **(B)** transfected with the indicated lentivirus. The percentage of SA-β-gal-positive cells counted in each condition was shown. The experiments shown are representative of three biological replicates. Data were compared by Student’s *t*-test; *P < 0.05, **P < 0.01. **(C,D)** Proliferation of 2BS and WI38 cells transfected with the indicated lentivirus measured by Cell Counting Kit-8 (CCK-8) assay. The absorbance at 450 nm was measured at 0–5 days. Data are shown as the mean ± SEM of the ratio for the absorbance. Data were compared by Student’s *t*-test, and data are shown as the mean ± SEM, with three independent experiments in each group; ***P < 0.005 vs. the NC group; ^##^*P* < 0.01, ^###^*P* < 0.005, Lenti-CMV-CTCF vs. pLenti-CMV-CTCF + shRNA-POLD1. **(E,F)** An 5-ethynyl-2′-deoxyuridine (EdU) incorporation assay was used to detect the rate of DNA synthesis. EdU-positive cells were quantified after 72 h of treatment by the ratio of the absorbance at 370 nm. Data were compared by one-way ANOVA and Student’s *t*-test, and data are shown as the mean ± SEM, with three independent experiments in each group; *P < 0.05, **P < 0.01. **(G,H)** Effects of altered CTCF expression on DNA damage in 2BS and WI38 cells transfected with the indicated lentivirus (magnification, × 100). The percentage of the tail area determined by CAPS software (*n* = 50). Data were compared by one-way ANOVA and Student’s *t*-test, and data are shown as the mean ± SEM. *P < 0.05, **P < 0.01.

To demonstrate the potential role of CTCF in cell senescence, the levels of senescence-associated β-galactosidase (SA-β-gal) staining, cell proliferation, DNA synthesis, and DNA damage were tested in 2BS and WI38 cells transfected with shRNA-CTCF, shRNA-CTCF + pLenti-CMV-POLD, pLenti-CMV-CTCF, or pLenti-CMV-CTCF + shRNA-POLD1. The results showed that the percentage of SA-β-gal-positive cells in 2BS and WI38 cells with CTCF knockdown was significantly increased relative to those of other groups, while the overexpression of POLD1 significantly attenuated the effects of shRNA-CTCF on aging. Accordingly, the percentage of SA-β-gal-positive cells in CTCF-overexpressing 2BS and WI38 cells was significantly decreased relative to that in other groups, and the percentage of positive cells was upregulated with the knockdown of POLD1 (Figures 6A,B).

The results of the Cell Counting Kit-8 (CCK8) assay showed that the proliferation of 2BS and WI38 cells transfected with shRNA-CTCF decreased significantly compared with that of the other groups of cells, and the proliferative capacity was restored with POLD1 overexpression. Cells transfected with pLenti-CMV-CTCF showed a significantly increased proliferative capacity compared with other groups of cells, and a decrease in proliferative capacity was observed in cells with POLD1 downregulation (Figures 6C,D).

The 5-ethynyl-2′-deoxyuridine (EdU) incorporation rate was quantified. The rate of EdU incorporation was lower in cells with CTCF knockdown than that in other groups of cells, and the rate was raised in cells with coinfected pLenti-CMV-POLD1. The rate of EdU incorporation was higher in cells overexpressing CTCF than in other groups of cells, and the rate decreased in cells coinfected with shRNA-POLD1 (Figures 6E,F). These results were consistent with the outcome of the CCK-8 assay, which indicated that CTCF promoted cell growth and proliferation through the POLD1 gene. DNA lesions were assessed using a single-cell gel electrophoretic assay (comet assay) under alkaline conditions. H_2_O_2_ can damage DNA (Zivkovic et al., 2019). The percentage of the comet tail area represents the level of DNA breakage. DNA damage levels were higher in the normal control, shRNA-CTCF, shRNA-CTCF + pLenti-CMV-POLD1, pLenti-CMV-CTCF, and pLenti-CMV-CTCF + shRNA-POLD1 cells when 2BS and WI38 cells were treated with H_2_O_2_. Notably, the DNA damage level in 2BS and WI38 cells transfected with shRNA-CTCF increased significantly, and this effect was reversed by POLD1 overexpression. We also found that CTCF overexpression in 2BS and WI38 cells significantly decreased the DNA damage level when cells were treated with H_2_O_2_, while the DNA damage level increased with the knockdown of POLD1 (Figures 6G,H). These results suggest that CTCF elevates DNA damage repairability, which could be attributed to POLD1 upregulation.

## Discussion

Aging refers to the phenomenon that the body’s physiological and psychological adaptability to the environment gradually decreases and tends to die, which is characterized by a series of specific changes in cellular morphology and senescence-associated genes (Pazolli and Stewart, 2008). Aging in cells is defined as a loss of replicative capacity, an arrest of the cell cycle, a shortened telomeric length, evident alterations of aging-related genes, and the appearance of aging phenotypes (McHugh and Gil, 2018). A decline in DNA synthesis and an increase in DNA breakage represent the most damaging DNA injuries that can compromise genomic integrity and viability, which are the main features of senescent cells (Gea et al., 2020).

POLD1, which is a central mediator of DNA replication and repair, plays an essential role in senescence to regulate the cell cycle and DNA damage repair. Some studies have shown that POLD1 gene mutation and expression were associated with the pathogenesis of Werner syndrome and MDPL (mandibular hypoplasia, deafness, progeroid features, and lipodystrophy) syndrome (Reinier et al., 2015; Fiorillo et al., 2018). CTCF is a TF that functions in many nuclear processes, including genomic organization, transcriptional regulation, insulator activity, and homologous recombination (HR)-mediated repair (Shan et al., 2019). CTCF mutations in humans are linked to microcephaly and intellectual disability (Gregor et al., 2013). Importantly, some findings identify the critical role of CTCF in selecting the homologous recombination repair pathway and in maintaining proper telomere replication and chromosome stability to participate in the progression of aging (Beishline et al., 2017; Hwang et al., 2019). In addition, CTCF could collaborate with Cockayne syndrome group B protein (CSB) to protect cells from oxidative stress and therefore delay aging (Lake et al., 2016). CTCF could also bind with the human longevity gene forkhead box O3 (FOXO3) and bind close to the peak of age-associated DNA changes and thereby participate in aging progression (Badarinarayan et al., 2017; Flachsbart et al., 2017; Han et al., 2020). In the present study, we successfully showed that CTCF, when binding to the POLD1 promoter, regulates the aging process.

First, we used the ENCODE database to predict whether CTCF binds to POLD1 in IMR90 cells. As shown in Figure 1, the more activated chromatin open region labeled with H3K27Ac, the denser the binding site of CTCF on the POLD1 promoter, which is consistent with the traditional view that TFs usually bind to DNA motifs in open chromatin regions (Zhu et al., 2016). The JASPAR database was also used to predict specific binding sites of CTCF in the POLD1 promoter, and five higher score sites were chosen to be verified by ChIP-qPCR. The results showed that CTCF bound mainly in the regions from −1,015 to −997 (site 3) and −625 to −607 (site 4) in the POLD1 promoter (Figure 1C). Furthermore, the activity of the POLD1 promoter was enhanced with the transfection of CTCF, and sites 3 and 4 played critical roles in the regulation of POLD1 by CTCF. These results indicated that CTCF could play a positive role in the transcriptional regulation of POLD1.

The age-related decrease in CTCF and POLD1 expression, as well as in the positive relationship between CTCF and POLD1 expression levels, were also observed in 2BS and WI38 cells, human lymphocytes, and SAMP8 mice (Figure 2). Moreover, the binding level of CTCF to the POLD1 promoter was verified to be attenuated with aging in 2BS cells, WI38 cells, and human lymphocytes (Figure 3). The results above suggested that the decrease in CTCF expression could be responsible for POLD1 downregulation by attenuating the binding level in senescence.

To test this hypothesis, CTCF expression was knocked down or upregulated in this study. Overexpression of CTCF in cells increased POLD1 transcription, and this CTCF-induced POLD1 upregulation was blocked by actinomycin D, an inhibitor of transcription, a further proof that CTCF regulates POLD1 at the transcriptional level (Figure 4). Compared with those in the normal control, both the expression of POLD1 and the CTCF-binding level to the POLD1 promoter were confirmed to be increased with higher CTCF expression and decreased with lower CTCF expression (Figure 4). In addition, the expression of POLD1 was altered by cotransfection with shRNA-POLD1 or pLenti-CMV-POLD1 (Figure 5). These results illustrated that the CTCF-binding level to the POLD1 promoter was positively regulated by CTCF expression and fully proved that the decrease in CTCF-mediated transcription was attributed to POLD1 downregulation in aging by regulating the binding level of CTCF to the POLD1 promoter.

To investigate the effect of CTCF expression level on cellular senescence, SA-β-gal staining, cell proliferation, DNA synthesis, and DNA damage levels were measured in cells transfected with shRNA-CTCF, shRNA-CTCF + pLenti-CMV-POLD1, pLenti-CMV-CTCF, and pLenti-CMV-CTCF + shRNA-POLD1. A higher percentage of SA-β-gal-positive cells, a striking decrease in cell proliferation, a lower rate of EdU incorporation, and a notable increase in DNA damage under oxidative stress were observed in cells transfected with shRNA-CTCF compared with control cells. Moreover, the opposite change was observed in 2BS and WI38 cells transfected with pLenti-CMV-CTCF compared with the control cells. In addition, the effect of CTCF on aging was reversed by exogenously altered POLD1 expression (Figure 6). These results indicated that CTCF participates in the aging process by regulating POLD1 transcription, which is responsible for DNA synthesis and DNA damage repair. Further investigation found that metformin, as an antiaging drug (Glossmann and Lutz, 2019), can promote the expression of CTCF and POLD1. Moreover, the decreased level of p16^INK4a^, elevated DNA synthesis, and reduced SA-β-Gal-positive rates were observed in 2BS cells cultured with 4 mM metformin. These results demonstrated that metformin could delay replicative senescence as CTCF agonist, and CTCF delays replicative senescence as a key TF of POLD1 (Supplementary Figure S1).

**FIGURE 7:**
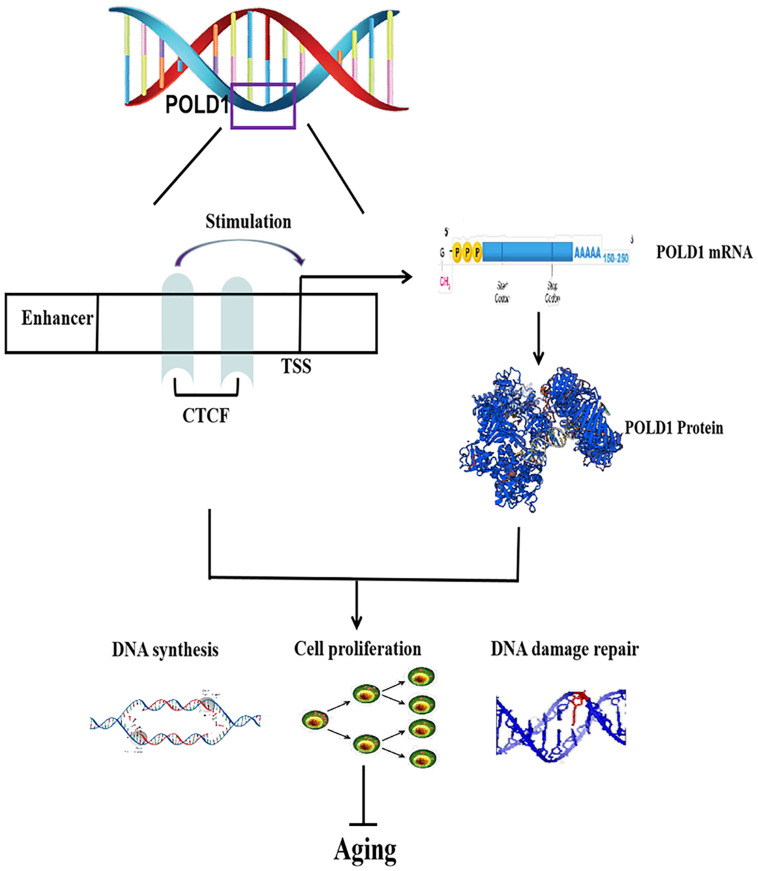
Regulatory mechanism of CCCTC-binding factor (CTCF) on aging. CTCF binds to the DNA polymerase delta 1, catalytic subunit (POLD1) promoter, and stimulates POLD1 expression to inhibit the progression of aging. TSS, transcription start site.

The role of POLD1 in aging may be regulated by multiple TFs or other factors (Gao et al., 2019), but our results propose the following POLD1 mechanism of aging: A decrease in POLD1 expression regulated by the decreased expression of the TF CTCF can accelerate aging by reducing cell proliferation, DNA synthesis, and DNA damage repair ability (Figure 7). As we first proved that CTCF expression levels reduced with aging and focused on the level of CTCF expression that affects the binding level of CTCF with the POLD1 promoter, it is worth to further explore if there are other factors that influence the binding affinity of CTCF with the POLD1 promoter.

## Materials and Methods

### Cell Lines and Culture Conditions

All cell lines, including 2BS and WI38, were purchased from the National Infrastructure of Cell Line Resource (Beijing, China). All cell lines were recently authenticated and tested for mycoplasma contamination when we purchased them. 2BS and WI38 cells were cultured in minimum essential medium (MEM; Gibco, Gaithersburg, MD, United States). All culture media were supplemented with 10% fetal bovine serum (FBS, Gibco), 100 U/ml penicillin, and 100 μg/ml streptomycin (Gibco), and cells were then cultured in a humidified incubator with 5% CO_2_ at 37°C. Mouse tail-tip fibroblasts (TTFs) were isolated from SAMP8 mice at different months of age and cultured at 37°C in Dulbecco’s modified Eagle’s medium (DMEM; Gibco) containing non-essential amino acids (Gibco) and 10% FBS (Gibco). Cell cultures were expanded through sequential subculturing using trypsin–EDTA (Gibco) to achieve a higher PD level.

### Animals

A total of 18 male SAMP8 mice were purchased from the First Affiliated Hospital of Tianjin University of Traditional Chinese Medicine (Tianjin, China). They were divided into three groups according to age: 2 months (2M, *n* = 6), 4 months (4M, *n* = 6), and 8 months (8M, *n* = 6). All animal experiments were approved by the Bioethics Committee of Xuanwu Hospital of Capital Medical University and complied with the National Institutes of Health Guide for the Care and Use of Laboratory Animals.

### Lymphocyte Isolation

Blood with anticoagulant EDTA-K_2_ was collected from volunteers who signed informed consent forms. The volunteers were recruited from the hospital staff, medical students, and healthy people from the health screening center of Xuanwu Hospital. Donors with cancer, blood disease, or recent inflammation were excluded. Eighty participants were enrolled in this study and divided into four age groups (in years): ages 20–29 (*n* = 20; mean age, 25), 40–49 (*n* = 20; mean age, 46), 60–69 (*n* = 20; mean age, 65), and 80–89 (*n* = 20; mean age, 86). Peripheral blood mononuclear cells were obtained by Ficoll-Paque (Solarbio, Beijing, China) density gradient centrifugation. The use of human specimens was approved by the appropriate institutional review boards.

### Bioinformatic Analysis

The 2,000-bp sequence upstream of the POLD1 transcription start site was obtained from the Ensembl genome browser^1^, and databases of ENCODE^2^ and JASPAR^3^ were used to predict the TFBS of CTCF on the POLD1 promoter. The binding sites predicted by the JASPAR database are shown in Table 1.

**TABLE 1:** Binding site prediction of CTCF in the POLD1 promoter region.

Model ID	Relative score	Start	End	Strand	Predicted site sequence
Site 1	0.78	−1,973	−1955	−1	CGACCAGGCGTGGGAGCCA
Site 2	0.77	−1,505	−1,487	−1	CAGCCGGGAAGGGTCAGTG
Site 3	0.77	−1,015	−997	−1	TGGCCCACAGGAGGCCTCA
Site 4	0.77	−625	−607	1	ATTCCAATCGAGGGCGAAA
Site 5	0.77	−136	−118	1	TTTCAGCAAGGGGGCGAGG

### Western Blotting

Briefly, total cellular and tissue proteins were extracted using radioimmunoprecipitation (Solarbio) assay buffer containing protease inhibitors (Solarbio). The protein concentration was determined using a BCA Protein Assay kit (Thermo Fisher Scientific, Massachusetts, United States). Then, 15 μg of cell or tissue lysate from each sample was separated by 8 or 12% sodium dodecyl sulfate (SDS) polyacrylamide gel electrophoresis. Electrophoretic transfer of proteins from gels onto nitrocellulose membranes was carried out in a trans-blotting chamber. Membranes were blocked by immersing in 5% non-fat milk (w/v) in TBST for 1 h to inhibit non-specific binding before being incubated with primary antibodies against CTCF (1:1,000; Abcam, Cambridge, United Kingdom), POLD1 (1:1,000; Abcam), and β-actin (1:5,000; Zhongshan Boil Tech Co., Beijing, China) at 4°C overnight. After rinsing with phosphate-buffered saline (PBS)/0.1% Tween-20, membranes were incubated with horseradish peroxidase-conjugated secondary antibodies (1:5,000; Zhongshan Boil Tech Co.). Immunocomplexes were visualized by incubation using an Enhanced Chemiluminescence Kit (Millipore, Massachusetts, United States). The bands were quantified with ImageJ software (National Institutes of Health, NIH, United States), and the target protein level was normalized to the β-actin level.

### RT-qPCR

Total RNA was isolated from cells and tissues with TRIzoL reagent (Thermo Fisher Scientific) and then reverse transcribed. RT-qPCR was performed using SYBR (Takara, Japan) on a Roche 480 machine (Basel, Switzerland). Quantification was performed by 2^–Δ^ ^Δ^ ^CT^ to calculate relative expression levels by subtracting the cycle threshold (CT) value of the control gene from the CT value of CTCF and POLD1. The primer sequences for PCR amplification of CTCF, POLD1, and β-actin are listed in Table 2.

**TABLE 2:** Antibodies and sequences used for Western blotting and RT-qPCR.

Protein	Mfrs./Made in	Cat no.
**Primary antibodies**		
CTCF	Abcam/UK	Ab188408
POLD1	Abcam/UK	Ab186407
β-actin	Zhongshan Boil Tech Co./China	TA-09
Goat anti-rabbit IgG	Abcam/UK	ab6721
**Secondary antibodies**		
HRP-conjugated anti-mouse IgG	Zhongshan Boil Tech Co./China	ZB2305
HRP-conjugated anti-rabbit IgG	Zhongshan Boil Tech Co./China	Zb5301
**Gene**		**Primer sequences**
β-actin	Forward	ACAGAGCCTCGCCTTTGC
	Reverse	CCACCATCACGCCCTGG
CTCF	Forward	TTCAGGTGGTTAAAGTGGGGGCCAA TGGAG
	Reverse	TCCTCTGTATAACGCAGTTTGCTCTTTTTG
POLD1	Forward	GCTCCGCTCCTACACGCTCAA
	Reverse	GGTCTGGTCGTTCCCATTCTGC
**ChIP assay promoter-specific qPCR**		
Site 1	Forward	AGCCAGGTCTAGTCCAAGCACAGAG
	Reverse	CGCAGGCTCACCAGCTTCATGA
Site 2	Forward	AGACCTATCGGCTCTCATCCCTTGG
	Reverse	GCGTGCTGGGCTTGAGGTGTAA
Site 3	Forward	GGACCTGGACTTCTGTCCGAACAAC
	Reverse	CCCGAGTCTTGGTAGGAGGATGGTT
Site 4	Forward	CGAAGCGACGAAGTTCCTCCAATCC
	Reverse	GCGCCCGGTACACTACTCTTAGGAT
Site 5	Forward	AGACCGCACGAGGTCGTGAAGGTA
	Reverse	CAAACAGCGTTTCCCGCCACAG

### Chromatin Immunoprecipitation Assay

ChIP assays were carried out using the ChIP assay kit (Thermo Fisher Scientific) according to the manufacturer’s protocol. 2BS and WI38 cells were fixed and processed for ChIP. Briefly, cells were rinsed with PBS before being cross-linked in a 1% formaldehyde solution for 10 min, and the cross-linking reaction was stopped by the addition of 2.5 M glycine to a final concentration of 0.125 M followed by an additional 5 min of gentle swirling. Cells were washed once with 4°C sterile PBS and then collected by adding 1 ml of 4°C sterile PBS containing protease inhibitors. Cells were scraped from the dish with a razor blade and transferred into a tube, which was centrifuged at 3,000 × *g* for 5 min at 4°C. Then, 1.0 μl of Micrococcal Nuclease (ChIP Grade) (10 U/μl) was added, and the tube was vortexed and incubated in a 37°C water bath for 15 min, mixing by inversion every 5 min. For immunoprecipitation, 10 μg of CTCF antibody (Abcam) and 2 μl of normal rabbit IgG (Abcam) were added to the sample, which was then incubated at 4°C with rotation for 12–16 h and washed according to the ChIP assay protocol. Cross-links were reversed on all samples, including 10% input according to the protocol. DNA was extracted from the digested samples using PCR purification columns following the manufacturer’s instructions (QIAGEN, Maryland). Then, 5 μl of the ChIP DNA sample was used in the subsequent 20 μl real-time PCR mixture. The primers used in the qPCR assessment are listed in Table 2. Normal rabbit IgG was used as negative control, and input was used as the positive amplification control, indicating 10% input DNA.

### Luciferase Reporter Assay

The 3′ untranslated region (UTR) of the POLD1 luciferase reporter plasmid (pHS-AVC-LW2301) was obtained from SyngenTech (Beijing, China). For the assessment of CTCF function on POLD1 promoter activity, genomic fragments harboring putative CTCF-binding sites in the human POLD1 promoter (∼1.8 kb upstream of the translation start site) were subcloned into the pGL4-Luc reporter vector (Promega) using Infusion 2.0 Dry-Down PCR cloning kit (Clontech, Shanghai, China). Promoter activity was further validated by mutation of the putative CTCF-binding site on the promoter at −1,015 to −997 or −625 to −607 by selecting these sites. For the luciferase reporter assay, 293T cells were seeded in a 24-well plate and incubated for 24 h before transfection. Subsequently, luciferase constructs, POLD1, and POLD1 + CTCF were co-transfected into 293T cells using Lipofectamine 3000. Cells were collected at 48 h after transfection and measured using the Dual-Luciferase Reporter System (Promega, WI, United States), according to manufacturer’s protocols. Three independent experiments were performed, and data were presented as mean ± *SD*.

### Lentivirus Transfection

To investigate the function of CTCF, we transduced pLenti-CMV-CTCF vector lentiviral constructs to overexpress CTCF in 2BS and WI38 cells. The empty pLenti-CMV vector served as a negative control. shRNA targeting CTCF (shRNA-CTCF) was used to knock down CTCF expression, and the negative control shRNA was purchased from GeneChem (Shanghai, China). We also purchased shRNA-POLD1 to knock down the expression of POLD1 and pLenti-CMV-POLD1 to overexpress POLD1 from GeneChem. 2BS and WI38 cells were infected with shRNA, shRNA-CTCF, empty pLenti-CMV, shRNA-POLD1, pLenti-CMV-CTCF, shRNA-POLD1, and pLenti-CMV-POLD1. The supernatant was removed after 16 h and replaced with fresh culture medium.

### Senescence-Associated (β-Galactosidase Staining

SA-β-gal (Beyotime, Beijing, China) activity was determined 72 h after transfection with lentivirus. Briefly, cells were washed with PBS and fixed for 15 min. Then, cells were washed three times with PBS and stained with X-gal solution for 6–24 h at 37°C. The population of SA-β-gal-positive cells was determined by counting 100 cells per dish, and images were taken using a phase-contrast microscope at 100× magnification (Olympus, Japan). The proportions of cells positive for SA-β-gal activity are presented as a ratio of the number of positive cells to the total number of cells counted in each dish. The results are expressed as the mean of triplicates ± SD.

### Cell Counting Kit-8 Assay

The proliferation of 2BS and WI38 cells was tested using CCK-8 kit (Beyotime). Briefly, cells were seeded in 96-well plates at 5 × 10^3^ cells/well with 100 μl of complete culture medium. At 0, 1, 2, 3, 4, and 5 days after adhesion, 10 μl of CCK-8 solution was added to each well, and the plates were incubated at 37°C for 2 h. The absorbance was measured at 450 nm using a microplate reader (Thermo Fisher Scientific).

### 5-Ethynyl-2′-Deoxyuridine Incorporation Assay

The DNA synthesis rate was assessed using the Beyo-Click^TM^ EdU kit (Beyotime). The cells were incubated with 10 μM EdU for 2 h, followed by staining according to the manufacturer’s instructions. The absorbance values of all wells at 370 nm were determined with an enzyme immunoassay instrument (Thermo Fisher Scientific).

### Comet Assay

Comet assays were used to examine 2BS and WI38 cells subjected to oxidative DNA damage under different conditions. At 72 h after transfection with CTCF overexpression lentivirus/shRNA-CTCF/shRNA-POLD1/pLenti-CMV-POLD1, the cells were treated with 100 μM H_2_O_2_ for 5 min at 4°C in the dark. Alkaline neutral comet assays were performed using a comet assay kit (Trevigen, Gaithersburg, MD, United States) according to manufacturer’s protocol. The mixture was loaded on the slide. The slide was immersed in an ice-cold lysis solution at 4°C for 60 min, and then alkaline unwinding solution was added for 60 min at 4°C. Electrophoresis was performed at 21 volts for 30 min in alkaline electrophoresis solution. After electrophoresis, the slides were washed with deionized H_2_O and then stained with SYBR Gold (1:10,000 dilution) for 30 min. A fluorescence microscope (OLYMPUS, Japan) was then used to observe the state of the cells. The percentage of the tail area of each cell was analyzed using CASP software (version 1.2.3, download in http://casplab.com/).

### Statistical Analysis

Statistical examination was carried out using GraphPad Prism version 8 (GraphPad Software, La Jolla, CA, United States). All data are displayed as mean ± *SD* (standard deviation). The difference between two groups was analyzed with a two-tailed *t-*test with variance equality. Differences among more than two groups were analyzed by one-way analysis of variance (ANOVA). CCK-8 data were analyzed by two-way ANOVA with repeated measures. The relationship between CTCF and POLD1 expression was calculated by Spearman’s rho test. *P* < 0.05 was considered to indicate a statistically significant result.

## Data Availability Statement

The original contributions presented in the study are included in the article/Supplementary Material, further inquiries can be directed to the corresponding author/s.

## Ethics Statement

The studies involving human participants were reviewed and approved by the Bioethics Committee of Xuanwu Hospital of Capital Medical University. The patients/participants provided their written informed consent to participate in this study. The animal study was reviewed and approved by the Bioethics Committee of Xuanwu Hospital of Capital Medical University.

## Author Contributions

YH and PW conceived the study. YH conducted the experiments. QS and SG performed the bioinformatics analysis. XZ conducted the data analysis. YW drafted the report. LJ and MC provided statistical support. JF and PW provided funding support. All authors interpreted the data and contributed to the final version of this report.

## Conflict of Interest

The authors declare that the research was conducted in the absence of any commercial or financial relationships that could be construed as a potential conflict of interest.

## Footnotes

1 http://www.ensembl.org/

2 https://www.encodeproject.org/

3 http://jaspar.binf.ku.dk/

## Supplementary Material

Supplementary Figure 1 Metformin could act as agonist of CTCF to delay cell senescence. **(A)** Protein expression of CTCF, POLD1, and p16^INK4a^ in 2BS cells treated with 4 mM metformin for 24 h. **(B)** SA-β-gal staining of 2BS cells treated with 4 mM metformin for 24 h. **(C)** EdU-positive cells were quantified after 24 h of treatment by the ratio of the absorbance at 370 nm. Data were compared by Student’s *t*-test, and data are shown as the mean ± SEM, with three independent experiments in each group. **P* < 0.05, ****P* < 0.001.

## Abbreviations

2BS: human embryonic lung diploid fibroblasts
ChIP: chromatin immunoprecipitation
CCK-8: Cell Counting Kit-8
EdU: 5-ethynyl-2 ′ -deoxyuridine
Pol δ: DNA polymerases delta
PD: population doubling
RT-qPCR: quantitative reverse transcription-polymerase chain reaction
SA- β-gal: senescence-associated β-galactosidase
SAMP8: senescence-accelerated mouse prone 8
shRNA: short hairpin RNA
TFBS: transcription factor binding site
WI38: human fetal lung fibroblasts
TTFs: mouse tail-tip fibroblasts.
