# Supplementary material for: CTCF Mediates Replicative Senescence Through POLD1
Source: Front Cell Dev Biol. 2021 Feb 22;9:618586. doi: 10.3389/fcell.2021.618586 (PMC7937641; doi:10.3389/fcell.2021.618586)
Supplement: Supplementary Figure 1 — Metformin could act as agonist of CTCF to delay cell senescence. (A) Protein expression of CTCF, POLD1, and p16INK4a in 2BS cells treated with 4 mM metformin for 24 h. (B) SA-β-gal staining of 2BS cells treated with 4 mM metformin for 24 h. (C) EdU-positive cells were quantified after 24 h of treatment by the ratio of the absorbance at 370 nm. Data were compared by Student’s t-test, and data are shown as the mean ± SEM, with three independent experiments in each group. *P < 0.05, ***P < 0.001. [file Data_Sheet_1.DOCX]

**Supplementary information**


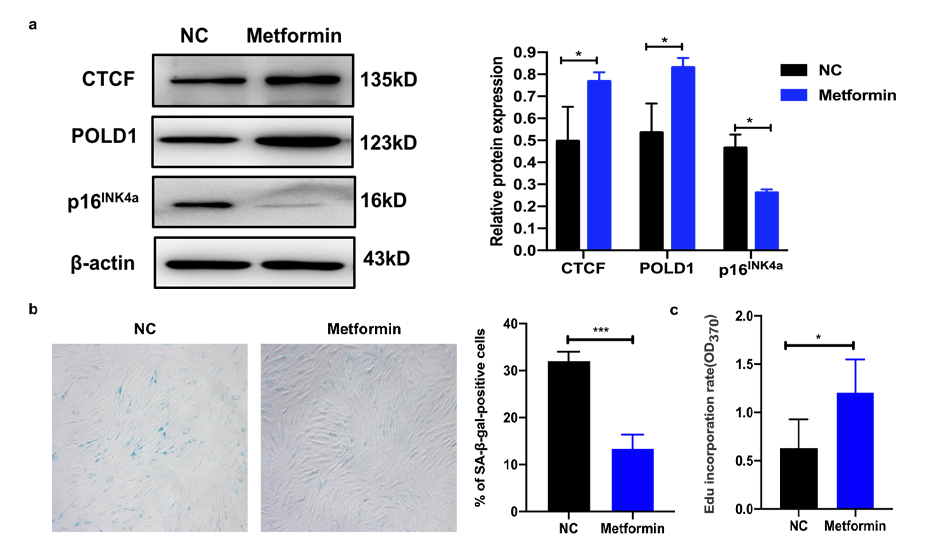


**Figure. S1 Metformin could act as agonist of CTCF to delay replicative senescence. a** The protein expression of CTCF, POLD1 and p16^INK4a^ in 2BS cells treated with 4mM metformin for 24h. **b** SA-β-gal staining of 2BS cells treated with 4mM metformin for 24h. **c** EdU-positive cells were quantified in the 2BS cells incubated with metformin for 24h by the ratio of the absorbance at 370 nm. The data was compared by Student’s t test, and the data are shown as the mean±SEM, with three independent experiments in each group; *P<0.05, ***P<0.001.
